# Language Proficiency and Sustained Attention in Monolingual and Bilingual Children with and without Language Impairment

**DOI:** 10.3389/fpsyg.2017.01241

**Published:** 2017-07-21

**Authors:** Tessel Boerma, Paul Leseman, Frank Wijnen, Elma Blom

**Affiliations:** ^1^Department of Special Education, Utrecht University Utrecht, Netherlands; ^2^Utrecht Institute of Linguistics OTS, Utrecht University Utrecht, Netherlands

**Keywords:** language impairment, bilingualism, sustained attention, vocabulary, morphology

## Abstract

**Background:** The language profiles of children with language impairment (LI) and bilingual children can show partial, and possibly temporary, overlap. The current study examined the persistence of this overlap over time. Furthermore, we aimed to better understand why the language profiles of these two groups show resemblance, testing the hypothesis that the language difficulties of children with LI reflect a weakened ability to maintain attention to the stream of linguistic information. Consequent incomplete processing of language input may lead to delays that are similar to those originating from reductions in input frequency.

**Methods:** Monolingual and bilingual children with and without LI (*N* = 128), aged 5–8 years old, participated in this study. Dutch receptive vocabulary and grammatical morphology were assessed at three waves. In addition, auditory and visual sustained attention were tested at wave 1. Mediation analyses were performed to examine relationships between LI, sustained attention, and language skills.

**Results:** Children with LI and bilingual children were outperformed by their typically developing (TD) and monolingual peers, respectively, on vocabulary and morphology at all three waves. The vocabulary difference between monolinguals and bilinguals decreased over time. In addition, children with LI had weaker auditory and visual sustained attention skills relative to TD children, while no differences between monolinguals and bilinguals emerged. Auditory sustained attention mediated the effect of LI on vocabulary and morphology in both the monolingual and bilingual groups of children. Visual sustained attention only acted as a mediator in the bilingual group.

**Conclusion:** The findings from the present study indicate that the overlap between the language profiles of children with LI and bilingual children is particularly large for vocabulary in early (pre)school years and reduces over time. Results furthermore suggest that the overlap may be explained by the weakened ability of children with LI to sustain their attention to auditory stimuli, interfering with how well incoming language is processed.

## Introduction

There is enormous variation in children’s rates and courses of language development, caused by the interplay of child-internal factors with a genetic basis (Stromswold, 2001), and child-external factors in the environment (Hoff, 2006). Child-internal and child-external factors can influence language outcomes in comparable ways, as is illustrated by the partially overlapping language profiles of children with an inborn primary or specific language impairment (further on called LI) and children who are raised bilingually. Profound language delays have been documented for both children with LI (Rice, 2004; Krok and Leonard, 2015; Rice and Hoffman, 2015) and bilingual children (Bialystok et al., 2010; Farnia and Geva, 2011; Paradis et al., 2016), and comparisons of these two groups of children showed strikingly similar performance on core language domains, such as vocabulary and morphology (Grüter, 2005; Paradis, 2005; Blom and Boerma, 2017). It is, however, unknown whether these similarities are temporary and limited to certain developmental stages. The first aim of the present study was therefore to compare the effects of LI and bilingualism on children’s language skills over time.

The second aim of the current study was to better understand why the language profiles of children with LI and bilingual children show overlap, so as to shed light on the underlying causes of the effects of LI on children’s language proficiency. Although the origins of the language delays are evidently different for the two groups of children, language input may play a key role in both. The quantity of language input is one of the most important factors contributing to the acquisition of language (Hart and Risley, 1995; Hoff, 2006) and it is well-established that the language outcomes of bilingual children are affected by the distributed nature of their input over two (or more) languages (e.g., Hoff et al., 2012). The language skills of children with LI may be poor due to an impaired capacity to process language input efficiently (e.g., Leonard et al., 2007b). Deficits in domain-general cognitive mechanisms are thought to underlie this limited input processing capacity, and particularly working memory has been frequently associated with the language difficulties of children with LI (for reviews, see Montgomery et al., 2010; Henry and Botting, 2016). There are furthermore intuitive and empirical reasons to assume interaction between language acquisition and attention mechanisms (Yoshida et al., 2011; Kapa and Colombo, 2014), which are tightly connected to working memory (Cowan, 1995; Baddeley, 2000), but less is yet known about this relation in children with LI.

A conceivable hypothesis is that the language problems of children with LI reflect a weakened ability to maintain attention to the stream of linguistic information, leading to incomplete processing of language input. In light of findings showing that children with LI have poor sustained attention (Ebert and Kohnert, 2011), the current study tested this hypothesis within a monolingual and bilingual context. We investigated the effects of LI and bilingualism on children’s auditory and visual sustained attention skills, and explored the role of sustained attention in explaining the effects of LI on children’s language outcomes. We hereby aimed to elucidate the relation between the linguistic and non-linguistic deficits of children with LI, which is a necessary step in further understanding the nature of the disorder (Kapa and Plante, 2015). Below, we first review research on the language development of bilingual children and children with LI, and discuss possible origins of their language delays. Subsequently, the relation between language and sustained attention is addressed. Throughout, we focus on the domains of vocabulary and morphology, as these are both considerably affected by LI and reduced input due to bilingualism (e.g., Blom and Boerma, 2017), and are subject of investigation in the present research.

It is well-documented that children who learn two or more languages, either from birth or later in childhood, lag behind their monolingual peers when only one of their languages is evaluated (Thordardottir et al., 2006; Scheele et al., 2010; Hoff et al., 2012). In early stages of acquisition, bilingual toddlers show slower rates of language-specific growth than monolingual toddlers, particularly in the domain of vocabulary which has been studied most often (Vagh et al., 2009; Silvén et al., 2014), but also in terms of grammar knowledge (Hoff et al., 2012). The consequent delays appear persistent, as is demonstrated by longitudinal research with bilingual (pre)schoolers (Farnia and Geva, 2011; Paradis et al., 2016). Tracking children’s vocabulary growth in English from grade 1 to 6, Farnia and Geva (2011) observed that their bilingual participants who learned English as a second language did not fully catch up with the monolingual controls, even though the bilinguals had a steeper learning curve in the primary grades and thus seemed to benefit from the increasing exposure to English at school. These findings correspond to results from other studies which indicate persistent gaps between the language-specific vocabulary size of monolingual and bilingual children (Appel and Vermeer, 1998; Cobo-Lewis et al., 2002; Roessingh and Elgie, 2009; Bialystok et al., 2010; Scheele, 2010; Thordardottir and Juliusdottir, 2013).

With respect to morphology, Paradis et al. (2016) also showed large and consistent delays over time, comparing bilingual children with monolingual norms. Around 60% of their Chinese-English participants did not achieve monolingual-like performance on an English verb morphology task after 6½12 years of English schooling (see also, Jia and Fuse, 2007), and, in addition, growth curves suggested plateau effects. Low saliency in the input may render verb morphology notoriously difficult for children learning English as a second language, as Paradis et al. (2016) suggest. Moreover, English verb inflection can be extra challenging for children who cannot benefit from the presence of tense and agreement morphology in their first language, like children with a Chinese background (Paradis, 2011; Blom et al., 2012). Using the same participant sample as Paradis et al. (2016) but including more general standardized measures of English vocabulary and grammar knowledge, Paradis and Jia (2017) reported monolingual-like attainment for the majority of children on the majority of measures after 5½12 years of English schooling. The persistence of bilingual children’s language delays may thus, next to language background, depend on linguistic subdomain.

Paradis and Jia (2017) furthermore found that children’s language environment, including amount and richness of English input, predicted their language abilities and convergence to monolingual norms. These findings connect to a multitude of studies which established that the amount and quality of language-specific input is a strong determinant of skills in that language (see, Hart and Risley, 1995; Huttenlocher et al., 2002; Rowe, 2012; Grüter and Paradis, 2014), and the distributed nature of bilingual children’s input is thereby one of the most important explanations for their documented language delays (Scheele et al., 2010; Hoff et al., 2012). Children’s scores on measures of vocabulary (Scheele et al., 2010; Chondrogianni and Marinis, 2011; Hoff et al., 2012) and morphology (Paradis, 2010a; Blom et al., 2012; Thomas et al., 2014) have both been related to amount of exposure, but there are indications that certain morphological structures are less susceptible to input effects than vocabulary (Chondrogianni and Marinis, 2011). Lexical items need to be learned one-by-one and can thus only be successfully acquired through repeated exposure to the same form. In contrast, (regular) morphology is largely based on rule learning and allows for fast generalization to new forms. This makes morphology possibly less sensitive to limited exposure, and thus bilingualism, than vocabulary, although bilingual performance also highly depends on other factors, such as the frequency and complexity of linguistic structures (Paradis, 2010a; Rispens and de Bree, 2015). In particular, structures that are low in frequency and high in complexity may be strongly influenced by reduced input.

An inborn LI disproportionately affects a child’s ability to learn language, in the absence of any clearly discernable cause (Leonard, 2014). Vocabulary is one domain in which delays are found (Rice and Hoffman, 2015), but LI is often more strongly associated with severe grammar weaknesses, especially in the domain of morphology (e.g., Rice et al., 1998; Ullman and Pierpont, 2005). Longitudinal work by Rice (2004, 2012) and Rice and Hoffman (2015) indicates that the delayed onset of language, characteristic of children with LI, is typically larger for grammar than for vocabulary. Once underway, both the lexical and grammatical development of children with LI seem to parallel the development of typically developing (TD) children.

Rice and Hoffman (2015) modeled the growth trajectories of children’s receptive vocabulary over nearly two decades. A consistently lower level of performance for the children with LI in comparison with their TD peers was found, but both groups had a generally similar growth curve. Only in the pre-adolescent period, rate of acquisition decelerated in children with LI. Similar growth patterns for children with TD and LI were also reported for measures of grammatical development, including the production and grammatical judgment of finiteness markings (Rice, 2012). The children with LI eventually reached, much later than TD peers, adult-like ceiling performance for production, but the more difficult judgment task remained problematic into adolescence. These findings from research by Rice and colleagues are in agreement with other large-scale longitudinal work with children with LI which showed persistent language delays and stability of growth in this population (Beitchman et al., 1996; Johnson et al., 1999), with differences in initial severity determining long-term language outcomes (Law et al., 2008; Conti-Ramsden et al., 2012). Moreover, these findings also correspond to recent work by Paradis et al. (2017) who compared the acquisition of tense morphology over time by bilingual children with and without LI, indicating developmental trajectories parallel to monolinguals with and without LI.

Several theories have been postulated to explain these persistent language delays of children with LI (see, Leonard, 2014). The current study, aiming to better understand the overlap between the language profiles of children with LI and bilingual children, will focus on accounts of LI that view the disorder as a problem of input or information processing (Kail, 1994; Leonard et al., 1997, 2007b). While factors in a child’s social context, like bilingualism, produce variation in the language input of a child, in turn influencing the child’s language development, it may be that an inborn LI leads to differences in how children can make use of the input. This hypothesis is based on findings from a growing body of work which suggests that problems of children with LI extend beyond linguistic domains (e.g., Henry et al., 2012; Vissers et al., 2015). Studies within the limited processing capacity framework have tried to integrate the linguistic and non-linguistic weaknesses of children with LI. Deficits in cognitive and perceptual mechanisms that are important for the acquisition of language, such as memory (Gathercole, 2006; Leonard et al., 2007b; Montgomery et al., 2010; Conti-Ramsden et al., 2015), and/or general speed of processing (Miller et al., 2001; Leonard et al., 2007b), may lead to incomplete or inadequate processing of the input, resulting in persistent language delays. As “cases of incomplete processing are assumed to be the functional equivalent of reductions in input frequency” (Leonard, 2014; p. 289), children with LI would need more exposure than their TD peers to successfully acquire language. This hypothesis is confirmed by several studies within the context of word learning (Rice et al., 1994; Gray, 2003; Riches et al., 2005; for a meta-analysis, see Kan and Windsor, 2010), and is furthermore supported by research on grammar acquisition showing that the effect of LI is more pronounced on low frequency than high frequency structures (Leonard et al., 2007a; Leroy et al., 2013).

A number of studies investigated the implications of these input dependencies for the language outcomes of bilingual children with LI, who are assumed to have a weaker capacity to process input efficiently compared with TD children, in addition to receiving less exposure in each language compared with monolingual children. Research conducted in the Netherlands showed that bilingual children with LI performed weaker on Dutch vocabulary and morphology tasks relative to both bilingual TD children and monolingual children with LI, indicating double delays (Verhoeven et al., 2011; Blom and Boerma, 2017). While the effect of LI on vocabulary scores was even larger in a bilingual than in a monolingual group of children, difficulty with morphology was not aggravated by the presence of LI in combination with bilingualism (Blom and Boerma, 2017; see also, Paradis, 2010b). Together with work that did not identify a double delay of bilingual children with LI on morphology (Paradis, 2007; Gutiérrez-Clellen et al., 2008; Rothweiler et al., 2012; Blom et al., 2013; Paradis et al., 2017), this supports the possibility that morphology is less susceptible to input effects than vocabulary (Chondrogianni and Marinis, 2011). However, the mixed findings within the domain of morphology also indicate that input effects may not always function linearly (Conti-Ramsden, 2010) and, in addition, that other factors are likely to play a role in explaining the performance patterns of bilingual children with LI, including the type of target structure and the characteristics of the bilingual sample (Gathercole, 2010; Paradis, 2010b).

Within the limited input processing capacity framework, working memory has been most frequently studied to account for the language difficulties of children with LI. There is substantial evidence for working memory problems in children with LI and several studies have found associations between working memory and language, pointing to a possible and plausible cause of the weakened language skills of these children (for a recent review, see Henry and Botting, 2016). Next to working memory, the role of attention resources in children with LI is a focus of recent research. Attention is a basic cognitive capacity which is difficult to reduce to a single definition. It can refer to a person’s ability to be alert, maintain focus over time, and selectively process relevant stimuli (Gomes et al., 2000). Common conceptualizations of attention imply strong connections between attention and language learning (for a review, see Ebert and Kohnert, 2011). For example, attention may be needed to direct a learner’s focus to relevant linguistic stimuli in the input before they can be processed, and to maintain this focus in order to prevent reduced or incomplete processing of that input. Moreover, it has been hypothesized that the ability to engage and disengage attention at a fast pace is necessary for the processing of rapidly presented stimulus sequences (Hari and Renvall, 2001), which is characteristic of language input. Empirical support for the role of attention in language learning has been provided by several studies, associating attention mechanisms with artificial word learning (Yoshida et al., 2011; Kapa and Colombo, 2014) and speech processing (see, Stevens and Bavelier, 2012) in TD children. Together with the high comorbidity rate between children with LI and children with attention deficits (Tirosh and Cohen, 1998), this explains the interest to attention in the LI literature.

A growing body of work suggests that, next to having working memory deficits, children with LI also have a limited attention capacity compared with their TD peers, even in children without comorbid attention deficit (hyperactivity) disorder (Marton, 2008; Ebert and Kohnert, 2011). Children with LI have particularly often been found to perform poorly on tasks tapping into sustained attention (for a meta-analysis, see Ebert and Kohnert, 2011). There is strong evidence that children with LI have a weak ability to maintain their focus on auditory stimuli during a prolonged period of time (Noterdaeme et al., 2001; Dodwell and Bavin, 2008; Spaulding et al., 2008). In addition, problems with visual sustained attention have also been reported (Finneran et al., 2009), although the effects of LI are smaller in comparison with the auditory domain and findings are mixed (Ebert and Kohnert, 2011).

A number of studies also examined the relationship between the poor language and sustained attention skills of children with LI, finding positive associations. Work by Montgomery showed that auditory sustained attention accounted for more than 45% of the variance in the online sentence processing of children with LI (Montgomery, 2008), and correlated highly with simple and complex sentence comprehension (Montgomery et al., 2009). Moreover, both auditory and visual sustained attention were positively correlated with picture-naming performance of children with LI and TD (Jongman et al., 2016), and auditory sustained attention was furthermore found to be associated with story generation skills (Duinmeijer et al., 2012). Blom and Boerma (2016) also investigated narrative abilities and showed that the effect of LI on story generation was mediated by sustained attention, measured with an integrated auditory and visual continuous performance task (CPT). Finally, findings from two intervention studies by Ebert et al. (2012, 2014) suggest that a treatment program designed to improve the processing speed and sustained attention skills of children with LI positively influenced children’s language scores. These studies thus support the possibility that the language delays of children with LI reflect, at least in part, a weakened ability to maintain attention to the stream of linguistic information, interfering with how well language input is processed. The present study will extend this research and investigate the role of auditory and visual sustained attention in explaining the effect of LI on two core language domains, vocabulary and morphology, which are known to be affected by LI and by reduced input.

The current study will analyze this within both a monolingual and bilingual context. As of yet, few studies have examined sustained attention in bilingual children. Although bilingual children have been reported to outperform their monolingual peers on different attention tests, especially those involving conflict processing (e.g., Bialystok, 1999; Engel de Abreu et al., 2012), the so-called bilingual advantage is not ubiquitous (e.g., Duñabeitia et al., 2014) nor undisputed (Paap et al., 2015). A specific bilingual benefit on sustained attention in children has not yet been attested and the few adult studies reveal mixed findings (Bialystok et al., 2008; Krizman et al., 2012; Bak et al., 2014), emphasizing the need for further research. In addition, work on the relation between sustained attention and language in bilingual children with LI is sparse, only including the intervention studies of Ebert et al. (2012, 2014) with Spanish-English bilingual participants with LI. Like the work with monolingual samples (Montgomery, 2008; Montgomery et al., 2009; Duinmeijer et al., 2012; Blom and Boerma, 2016; Jongman et al., 2016), these studies suggest that sustained attention may also contribute to the language difficulties of children with LI growing up in bilingual learning settings. The current research will further explore this.

The first aim of the present study was to investigate whether the overlap between the language profiles of children with LI and bilingual children was temporary, or persisted over time. We used a four-group design, including monolingual and bilingual children with and without LI, which allowed for a systematic examination of the effects of LI and bilingualism on children’s language development. We focused on children’s vocabulary and morphology outcomes. Negative effects of LI were expected to emerge on both language domains (Krok and Leonard, 2015; Rice and Hoffman, 2015), although larger effects were anticipated on morphology (Rice, 2012). Given the young age of the participants (5–8 years old) and the relatively short time span of the current study (3 years), effects of LI were furthermore assumed to remain stable over time (Rice, 2012). Vocabulary and morphology were also predicted to be negatively affected by reductions in input frequency as a result of bilingualism (Hoff et al., 2012; Paradis et al., 2016), with possibly more pronounced effects on vocabulary than morphology (Chondrogianni and Marinis, 2011). The gap between the monolinguals and bilinguals was not expected to fully close within the time frame of this study, but the effect of bilingualism may diminish over time due to accumulating input in school (Farnia and Geva, 2011).

The second aim of the current study was to better understand why similarities between the language profiles of children with LI and bilingual children emerge. We tested the hypothesis that the language difficulties of children with LI stem from auditory sustained attention deficits, since consequent incomplete processing of language input may lead to delays that are comparable to those originating from reductions in input frequency due to bilingualism. Visual sustained attention was also assessed to examine possible domain-general origins. Furthermore, the hypothesis was tested within a monolingual and bilingual context. The presence of LI was predicted to impact children’s sustained attention skills, with relatively better performance of children with LI on the visual compared with the auditory domain (Ebert and Kohnert, 2011). Sustained attention was not hypothesized to be strongly influenced by bilingualism, although positive effects were considered possible in view of the literature on the cognitive benefit of bilingualism (e.g., Bialystok, 1999).

Previous work with children with LI showed that limitations in sustained attention are predictive of narrative skills (Blom and Boerma, 2016), and associated with sentence processing (Montgomery, 2008) and picture-naming (Jongman et al., 2016). We anticipated that sustained attention, and in particular auditory sustained attention, would also play a role in explaining the effect of LI on two core language areas, i.e., vocabulary and morphology, which are known to be influenced by a limited amount of input (e.g., Scheele et al., 2010; Blom et al., 2012) and thus likewise by the functional equivalent: incomplete processing of input. Given our hypothesis that the language delays of children with LI arise from a weakened ability to maintain attention to the stream of linguistic information, interfering with efficient input processing, effects of visual sustained attention were expected to be limited. Moreover, the impact of sustained attention deficits on morphology could be less pronounced in comparison with vocabulary, as previous work showed that morphology is less susceptible to input effects than vocabulary (Chondrogianni and Marinis, 2011). However, this may also depend on the frequency and complexity of the targeted structures (Paradis, 2010a; Rispens and de Bree, 2015). Finally, we had no clear theoretical or empirical reasons to assume substantial differences between the role of sustained attention in explaining the effect of LI on monolingual or bilingual children’s language skills.

## Materials and Methods

### Participants

The data from the current study were collected within a large-scale longitudinal project that aimed to investigate the linguistic and cognitive development of children with diverse language backgrounds in the Netherlands. Four groups, monolingual and bilingual children with and without LI, were followed from 2014 to 2016 and tested once a year (mean = 11 months). Children were around age 5 or 6 at the first wave of testing, and around age 7 or 8 at the third and last wave. For the present study, a matched subsample of this large-scale project was selected to be able to control for factors such as age, non-verbal intelligence (NVIQ) and socio-economic status (SES) when comparing different groups of children, as these factors may influence children’s language skills (Hart and Risley, 1995; Conti-Ramsden et al., 2012). The group of bilingual children with LI (BILI) was the smallest (*N* = 33) and therefore the basis for our participant match. Before wave 3, one child in the BILI group transferred to a school for children with an intellectual disability and was therefore excluded from the current study, resulting in groups of 32 children each (total *N* = 128). Each child in the BILI group was matched on age in months at wave 1 to a bilingual typically developing child (BITD), a monolingual typically developing child (MOTD), and a monolingual child with LI (MOLI). As the BILI group had a relatively large age range and was on average slightly older than the other groups, it was not possible to find a close age match (i.e., a difference of less than 4 months) for all children. Some children were therefore matched on group level, aiming to form groups that were on average as comparable as possible. To this end, groups were furthermore matched on (in order of priority) NVIQ, exposure to Dutch (for the bilinguals), SES, and gender.

Group characteristics are displayed in **Table 1**. There were no significant differences between the four groups of children in age in months at wave 1 [*F*(3,124) = 0.25, *p* = 0.86, $\eta_{p}^{2}$< 0.01], wave 2 [*F*(3,124) = 0.03, *p* = 0.99, $\eta_{p}^{2}$< 0.01], nor wave 3 [*F*(3,124) = 0.07, *p* = 0.98, $\eta_{p}^{2}$< 0.01]. NVIQ, measured with the short version of the *Wechsler Nonverbal-NL* (Wechsler and Naglieri, 2008), did not significantly differ between the groups of children either [*F*(3,124) = 1.02, *p* = 0.39, $\eta_{p}^{2}$= 0.02]. In addition, no differences emerged in SES [*H*(3) = 5.5, *p* = 0.14], which was indexed by the average education level of the child’s parents, measured on a nine-point scale (ranging from 1 ‘no education’ to 9 ‘university degree’). There were also no gender differences between the four groups of children [χ^2^(3, *N* = 128) = 6.4, *p* = 0.09], although there was a relatively large number of boys in the groups of children with LI. Finally, the bilingual groups did not significantly differ in exposure to Dutch before the age of 4 [*F*(1,61) = 0.68, *p* = 0.41, $\eta_{p}^{2}$= 0.01], nor current exposure to Dutch at home [*F*(1,62) = 2.5, *p* = 0.12, $\eta_{p}^{2}$= 0.04]. The *Questionnaire for Parents of Bilingual Children* (PaBiQ; Tuller, 2015), administered at wave 1, measured the exposure to Dutch before the age of 4 as the percentage of input in Dutch that the child received before this age (both inside and outside home context), relative to the total amount of language input. The PaBiQ measured current exposure to Dutch at home as the percentage of input in Dutch, relative to the total amount of language input, that the child heard from its mother, father, siblings, and other adults that had frequent contact with the child.

**Table 1:** Demographic characteristics of the participants.

		Age (months) Wave 1	Age (months) Wave 2	Age (months) Wave 3	Non-verbal IQ (standardized)	Socio-economic Status	Gender		Exposure to Dutch before age 4 (in %)	Exposure to Dutch at Wave 1 (in %)
	*N*	Mean (*SD*)	Mean (*SD*)	Mean (*SD*)	Mean (*SD*)	Mean (*SD*)	*N*_GIRLS_ (%)	*N*_BOY S_ (%)	Mean (*SD*)	Mean (*SD*)
MOTD	32	70.9 (7.0)	82.5 (6.9)	94.1 (6.9)	100.4 (11.9)	6.3 (2.0)	14 (44%)	18 (56%)	n/a	n/a
MOLI	32	71.4 (6.3)	82.8 (6.5)	94.6 (6.6)	97.5 (12.9)	5.2 (1.8)	8 (25%)	24 (75%)	n/a	n/a
BITD	32	71.3 (7.3)	83.0 (7.1)	94.8 (7.1)	95.8 (15.0)	5.3 (2.3)	17 (53%)	15 (47%)	43.0 (8.3)	50.9 (12.0)
BILI	32	72.4 (8.6)	83.0 (8.9)	94.7 (8.8)	94.7 (15.3)	5.7 (2.2)	10 (31%)	22 (69%)	40.9 (11.1)	45.2 (16.5)

*MOTD, monolingual typically developing; MOLI, monolingual language impaired; BITD, bilingual typically developing; BILI, bilingual language impaired.*

#### Criteria for LI

All children in the MOLI and BILI groups had been diagnosed with LI before the start of this research. They were diagnosed with LI by licensed clinicians according to the standardized criteria that are used in the Netherlands. In the Netherlands, a child officially meets the criteria for LI when (s)he obtains a score of at least 2 standard deviations (*SD*) below the mean on an overall score of a standardized language assessment test battery or a score of at least 1.5 *SD* below the mean on two out of four subscales of this standardized language assessment (Stichting Siméa, 2014). The most commonly used test batteries include the Dutch version of the *Clinical Evaluation of Language Fundamentals* (CELF-4-NL; Kort et al., 2008), the *Schlichting Test for Language Production and Comprehension* (Schlichting and Lutje Spelberg, 2010a,b), and the *Dutch Language Proficiency Test for All Children* which has bilingual norms [Taaltoets Alle Kinderen (TAK); Verhoeven and Vermeer, 2001]. In addition, a guideline focusing on the assessment of bilingual children is provided by Stichting Siméa (2016), stating the need for a bilingual anamnesis and, if possible, evaluation of the first and second language.

At wave 1 and 2, all 64 children in the MOLI and BILI groups met the criteria for LI that were specified above. At wave 3, eight children (four bilingual and four matched monolingual children) did not meet these criteria anymore, confirming the fluid developmental pathways for language (Reilly et al., 2014). Given their history of LI and the long-term persistence of the language problems (Scarborough and Dobrich, 1990), we did not exclude these children. All children who participated in the present study had no intellectual disability (NVIQ range from 70 to 130), hearing impairment, severe articulatory difficulties or diagnosed attention deficit disorder. At the start of the research, 63 children with LI attended special education and one child with LI attended regular education with ambulatory care. During the study, 14 children with LI (five bilingual and nine monolingual) transferred from special to regular education. All TD children attended regular elementary schools and did not have documented language problems.

#### Criteria for Bilingualism

Information about the home language environment of the children was provided by the parental questionnaire (PaBiQ; Tuller, 2015). A child was assigned to the monolingual group if both parents were native speakers of Dutch and always spoke Dutch to the child. A child was considered bilingual if at least one parent was a native speaker of another language than Dutch and spoke their mother tongue with the child for an extensive period of the child’s life. All bilingual children who participated in this study were born in the Netherlands and learned Dutch as a second language. As elementary school starts at age 4 in the Netherlands, all children had received at least approximately 1 year of schooling in Dutch before the first wave of testing. The first languages of the bilingual TD children included Turkish (*N* = 14), Tarifit-Berber (*N* = 10), and Moroccan Arabic (*N* = 8). The first languages of the bilingual children with LI were Turkish (*N* = 10), Moroccan Arabic (*N* = 7), Egyptian Arabic (*N* = 3), Tarifit-Berber (*N* = 2), Dari (*N* = 2), Chinese (*N* = 1), Pashto (*N* = 1), Suryoyo (*N* = 1), Kirundi (*N* = 1), Russian (*N* = 1), Portuguese (*N* = 1), Danish (*N* = 1), and Frisian (*N* = 1).

### Materials and Procedures

The current study was part of a large-scale project which was approved by the Standing Ethical Assessment Committee of the Faculty of Social and Behavioral Sciences at Utrecht University. Parents of participants signed an informed consent form. Children were individually tested in a quiet room at their school. Trained research assistants followed a strict protocol and administered a test battery, consisting of language, memory and attention tasks, in two separate sessions. Each test session lasted approximately 1 h. Receptive vocabulary, morphology and sustained attention were all assessed in the second session. Similar procedures were used at each wave of testing.

#### Language

Receptive vocabulary was tested at all three waves with the *Peabody Picture Vocabulary Test* (PPVT-III-NL; Schlichting, 2005), which is a standardized test designed for a wide age range (2;3–90 years). Participants hear a target word and have to pick the correct referent out of four pictures. The task is divided in 17 sets, which increase in difficulty, with 12 target words in each set. We administered the PPVT-III-NL according to the official guidelines and thus determined the starting set based on a child’s age. The task was terminated when a child picked the incorrect referent picture nine or more times in a set. Raw scores were used in the analyses.

Grammatical morphology was assessed at all three waves with a subtest of the *Dutch Language Proficiency Test for All Children* (TAK; Verhoeven and Vermeer, 2001), suitable for children aged 4–9. The subtest ‘Word Formation’ elicits 12 noun plurals and 12 past participles, including both regularly and irregularly inflected nouns and verbs. Children are presented with a picture and asked to finish an incomplete sentence uttered by the experimenter, hereby eliciting the plural of a noun (e.g., *Dit is één lepel, dit zijn twee…? Lepels.* [*This is one spoon, these are two…? Spoons*]) or the past participle of a verb (e.g., *Hier zie je Paul op de bank zitten. Gisteren heeft hij ook al op de bank…? Gezeten.* [*Here you see Paul sitting on the couch, yesterday he has also*… *on the couch*? *Sat.*]). Accuracy was scored offline by a native speaker of Dutch and the number of items correct (maximum = 24) was used in the analyses.

#### Sustained Attention

Sustained attention was measured at wave 1 with an integrated visual and auditory CPT, which was based on the IVA+Plus (Sandford and Turner, 2004) and identical to the task used in Blom and Boerma (2016). The task was administered on a laptop using the experimental software E-Prime 2.0 (Schneider et al., 2002). Children were presented with visual and auditory stimuli that could either be a target (number ‘1’) or a distractor (number ‘2’). Each visual stimulus was presented for 167 milliseconds. Irrespective of modality, children were asked to press the space bar in response to a target stimulus, but to refrain from responding when a distractor appeared. The test included 168 trials, excluding the practice phase, in which visual and auditory targets (*N* = 84) and distractors (*N* = 84) were mixed and presented randomly. The task lasted approximately 10 min, during which children were required to stay alert and maintain their attention.

Response sensitivity on this task was scored as *d*′ (Macmillan and Creelman, 2005). For visual sustained attention, this inherently dual score reflects percent correct responses to visual targets (hits) relative to percent incorrect responses to visual distractors (false alarms). For auditory sustained attention, correct and incorrect responses to auditory targets and distractors were used, respectively. By taking into account both hits and false alarms, this score controls for potential response bias, such as a child pressing the space bar in response to each stimulus. Correct responses to the target with a reaction time below 100 milliseconds were excluded (<1% of all trials). The *d*′ statistic is calculated as follows: *d*′ = z(hits) - z(false alarms). The higher the statistic, the better the child’s response sensitivity. Macmillan and Creelman (2005; p. 8) indicate that proportions correct between 0.6 and 0.9 roughly correspond to *d*′ values between 0.5 and 2.5.

### Data-Analysis

All statistical analyses were done with SPSS 22 (IBM Corp., 2013). Exploration of the data indicated that the dependent variables were normally distributed. NVIQ and SES were added as covariates in all analyses to ensure that these background variables could not influence the results. We first investigated the effects of LI and bilingualism on children’s language skills over time, and on their visual and auditory sustained attention measured at wave 1. A 3 × 2 × 2 mixed-design analysis of covariance (ANCOVA) was conducted for vocabulary and morphology scores separately. Time (Wave 1, 2, and 3) was included as within-subjects factor, and Language Group (monolingual, bilingual) and Impairment Status (TD, LI) as between-subjects factors. *Post hoc* tests were conducted in case significant interactions between the factors in the analyses were observed. For sustained attention, a multivariate ANCOVA included Impairment Status (TD, LI) and Language Group (monolingual, bilingual) as fixed factors and auditory and visual sustained attention as dependent variables. Given the difference in modality, we were hesitant to view the two dependent variables as part of one and the same construct and we thus opted for a MANCOVA instead of a mixed-design ANCOVA (both analyses, however, showed the same patterns).

**FIGURE 1:**
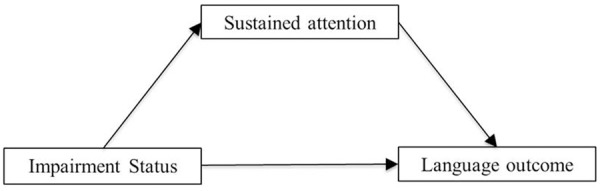
Mediation model.

Subsequently, mediation analyses in the monolingual and bilingual group separately were performed with the PROCESS application for SPSS of Hayes (2013), aiming to find relationships between Impairment Status (the independent variable X), sustained attention (the mediator M), and children’s language skills (the dependent variable Y). One important prerequisite of this model is that a cause must precede an effect in time. That is, a change in X must have time to affect a change in M, which, again, must have time to affect a change in Y. To meet the requirement of temporal precedence, we used children’s language outcomes at wave 2 and 3 as dependent variables, and sustained attention measured at wave 1 as mediator. The group distinction (TD-LI), which was the independent variable, was based on assessments prior to wave 1. A visual representation of the mediation model is depicted in **Figure 1**. Separate mediation analyses were done for each language domain at wave 2 and 3 to assess the stability of the effect, and for auditory and visual sustained attention, due to a high correlation between the two (*r* = 0.67, *p* < 0.001). To control for possible effects of language background, all analyses described above were also conducted for a subsample of the participants, excluding bilingual children with LI who had a different first language than the bilingual TD children. Analyses yielded similar results and are therefore not reported.

## Results

### Language Development

#### Vocabulary

**Table 2** presents the means and *SD*s of children’s performance on the PPVT-III-NL, measuring receptive vocabulary. Results revealed a significant main effect of Time [*F*(2,238) = 284.1, *p* < 0.001, $\eta_{p}^{2}$= 0.71], indicating that the vocabulary size of children increased over time, with significant differences across all three waves (all *p* < 0.001). Furthermore, significant main effects of Impairment Status [*F*(1,119) = 33.3, *p* < 0.001, $\eta_{p}^{2}$= 0.22] and Language Group [*F*(1,119) = 26.2, *p* < 0.001, $\eta_{p}^{2}$= 0.18] were found. Children with LI and bilingual children had lower vocabulary scores than TD and monolingual children, respectively. A significant interaction effect of Time × Language Group also emerged [*F*(2,238) = 3.1, *p* = 0.047, $\eta_{p}^{2}$= 0.03] and will be discussed below. Other interactions were not significant. Non-verbal IQ was a significant covariate [*F*(1,119) = 18.0, *p* < 0.001, $\eta_{p}^{2}$= 0.13], while SES was not.

**Table 2:** Dutch receptive vocabulary skills of the four groups of children (raw scores PPVT).

		PPVT (raw) Wave 1		PPVT (raw) Wave 2		PPVT (raw) Wave 3	
	*N*^a^	Mean (*SD*)	Range	Mean (*SD*)	Range	Mean (*SD*)	Range
MOTD	32	86.6 (10.4)	57–103	98.5 (9.6)	79–122	103.8 (8.9)	82–124
MOLI	32	76.5 (9.2)	54–93	86.6 (11.9)	62–110	95.9 (10.1)	73–114
BITD	32	75.8 (10.5)	57–97	87.8 (11.5)	64–104	96.2 (12.4)	70–125
BILI	32	62.9 (13.3)	33–86	77.8 (15.5)	53–106	86.3 (14.0)	58–114

*MOTD, monolingual typically developing; MOLI, monolingual language impaired; BITD, bilingual typically developing; BILI, bilingual language impaired; PPVT, Peabody Picture Vocabulary Task.*

*^a^For one child in the MOTD group and one child in the BITD group, raw PPVT scores at wave 1 were not available due to incorrect assessment procedures. Moreover, raw PPVT scores at wave 2 were not available for one child in the BITD group.*

*Post hoc* analyses were performed to unpack the interaction between Time × Language Group, which showed that the vocabulary size of both monolingual and bilingual children increased over time (all *p* < 0.001). Moreover, univariate ANCOVA’s testing group performance on the PPVT-III-NL at wave 1, 2, and 3 separately showed a significant effect of Language Group at each wave. The magnitude of the effect decreased, being large at Wave 1 and medium at Wave 2 and 3 (Wave 1: *p* < 0.001, $\eta_{p}^{2}$= 0.18; Wave 2: *p* < 0.001, $\eta_{p}^{2}$= 0.10; Wave 3: *p* = 0.001, $\eta_{p}^{2}$= 0.09). Thus, the difference in vocabulary size between the monolingual and bilingual children became smaller over time, but the gap was not fully closed.

#### Morphology

**Table 3** presents the means and *SD*s of children’s performance on the TAK Word Formation task, measuring grammatical morphology. Results revealed a significant main effect of Time [*F*(2,242) = 167.6, *p* < 0.001, $\eta_{p}^{2}$= 0.58], indicating that children’s performance on the word formation task improved over time, with significant differences across all three waves (all *p* < 0.001). In addition, a significant main effect of Impairment Status [*F*(1,121) = 65.8, *p* < 0.001, $\eta_{p}^{2}$= 0.35] and a significant main effect of Language Group [*F*(1,121) = 16.4, *p* < 0.001, $\eta_{p}^{2}$= 0.12] emerged. Children with LI and bilingual children had weaker morphological skills than TD and monolingual children, respectively. There were no significant interaction effects. Non-verbal IQ was a significant covariate [*F*(1,120) = 4.3, *p* = 0.04, $\eta_{p}^{2}$= 0.04], while SES was not.

**Table 3:** Dutch morphology skills of the four groups of children (raw scores TAK Word Formation).

		TAK Word Wave 1		TAK Word Wave 2		TAK Word Wave 3	
	*N*^a^	Mean (*SD*)	Range	Mean (*SD*)	Range	Mean (*SD*)	Range
MOTD	32	15.3 (3.8)	7–23	18.6 (3.5)	11–24	20.5 (3.6)	11–24
MOLI	32	10.0 (3.7)	2–17	12.3 (4.1)	4–22	15.5 (3.9)	8–23
BITD	32	11.7 (5.1)	0–20	15.3 (4.3)	6–21	18.1 (3.9)	10–24
BILI	32	6.5 (4.7)	0–15	11.0 (3.8)	0–19	12.7 (5.1)	3–24

*MOTD, monolingual typically developing; MOLI, monolingual language impaired; BITD, bilingual typically developing; BILI, bilingual language impaired; TAK, Taaltoets Alle Kinderen.*

*^a^For one child in the BILI group, raw TAK scores at wave 1 were not available due to a refusal to cooperate.*

### Sustained Attention

**Table 4** presents the performance per group on the CPT, split up for auditory and visual stimuli. A multivariate ANCOVA with CPT Auditory and CPT Visual as dependent variables and Impairment Status and Language Group as independent variables revealed a significant negative effect of Impairment Status [*F*(2,121) = 10.9, *p* < 0.001, $\eta_{p}^{2}$= 0.15], whereas there was no main effect of Language Group nor an interaction effect of Impairment Status × Language Group. Non-verbal IQ was a significant covariate [*F*(2,121) = 13.5, *p* < 0.001, $\eta_{p}^{2}$= 0.18], while SES was not. Bonferroni-corrected pairwise comparisons showed that children with LI scored more poorly on the auditory [*F*(1,122) = 11.2, *p* = 0.001, $\eta_{p}^{2}$= 0.08] as well as the visual [*F*(1,122) = 21.4, *p* < 0.001, $\eta_{p}^{2}$ine-formula>= 0.15] component of the CPT in comparison with their TD peers. Paired samples *t*-tests in each group separately indicated that both the monolingual TD children [*t*(31) = 2.6, *p* = 0.01, *d* = 0.28] and the monolingual children with LI [*t*(31) = 2.3, *p* = 0.03, *d* = 0.46] performed significantly better on the auditory stimuli than on the visual stimuli. There were no differences between the two components of the CPT in both bilingual groups. Below, mediation analyses investigating the role of auditory and visual sustained attention in explaining the effect of LI on the children’s language outcomes will be conducted separately for the monolingual and bilingual group of children.

**Table 4:** Performance on the sustained attention task (CPT d′).

		CPT (d′) Auditory		CPT (d′) Visual	
	*N*	Mean (*SD*)	Range	Mean (*SD*)	Range
MOTD	32	2.33 (0.9)	0.53–4.08	2.09 (0.8)	–0.05–3.67
MOLI	32	1.72 (1.0)	–0.27–3.84	1.31 (0.8)	0.14–3.63
BITD	32	2.05 (0.8)	–0.13–4.08	2.02 (0.9)	0.22–4.08
BILI	32	1.51 (1.0)	–0.34–3.41	1.36 (1.0)	–0.78–3.67

*MOTD, monolingual typically developing; MOLI, monolingual language impaired; BITD, bilingual typically developing; BILI, bilingual language impaired; CPT, Continuous Performance Task.*

#### Effect of LI in the Monolingual Group

**Table 5** presents the results of the mediation analyses investigating the effects of auditory and visual sustained attention on the relation between Impairment Status and language outcomes in the monolingual group of children. To determine whether the effect of Impairment Status on children’s language outcomes is significantly reduced due to sustained attention (i.e., the indirect or mediation effect), bootstrapped tests (5.000 – bias-corrected), and confidence intervals were used, as these are more reliable than *p*-values. Meaningful mediation is assumed if zero is not included in the confidence intervals of the indirect effects. The results indicate that auditory sustained attention mediated the effect of LI on both vocabulary at wave 2 and 3, and grammatical morphology at wave 2 and 3. At wave 2, the index of mediation (the standardized indirect effect) was slightly larger for vocabulary (*b* = -0.08, 95% CI [-0.22, -0.01]) than morphology (*b* = -0.05, 95% CI [-0.18, -0.001]), but there was substantial overlap in confidence intervals, indicating that reliable differences cannot be assumed. The index of mediation was the same for both domains at wave 3 (vocabulary: *b* = -0.07, 95% CI [-0.21, -0.002]; morphology: *b* = -0.07, 95% CI [-0.19, -0.01]). Although auditory sustained attention significantly reduced the effect of Impairment Status on children’s language outcomes, it only accounted for part of the relationship. The direct effect of Impairment Status on children’s language outcomes remained significant when auditory sustained attention was controlled for. Results furthermore showed that visual sustained attention was not a meaningful mediator, as it did not significantly reduce the relation of X on Y. Correlations between children’s language and sustained attention skills and visual representations of the mediation models are provided in the Supplementary Table 1 and Figures 1–4, respectively.

**Table 5:** Mediation effects of auditory and visual sustained attention on the relation between Impairment Status and language outcomes in the monolingual group of children.

			Auditory			Visual		
				95% CI			95% CI	
Language outcome	Wave	Effect	*b*	Lower	Upper	*b*	Lower	Upper
Vocabulary	2	Total effect	–10.75	–16.27	–5.23	–10.75	–16.27	–5.23
		Direct effect	–8.79	–14.29	–3.30	–9.45	–15.53	–3.36
		*Indirect effect*	–***1.96***	–***5.78***	–***0.14***	–*1.31*	–*5.23*	*1.16*
	3	Total effect	–6.31	–11.03	–1.59	–6.31	–11.03	–1.59
		Direct effect	–4.94	–9.73	–0.16	–4.05	–9.11	1.01
		*Indirect effect*	–***1.36***	–***4.67***	–***0.04***	–*2.26*	–*6.59*	*0.01*
Morphology	2	Total effect	–5.83	–7.82	–3.85	–5.83	–7.82	–3.85
		Direct effect	–5.32	–7.35	–3.29	–5.30	–7.48	–3.12
		*Indirect effect*	–***0.51***	–***1.74***	–***0.01***	–*0.53*	–*1.79*	*0.26*
	3	Total effect	–4.51	–6.39	–2.63	–4.51	–6.39	–2.63
		Direct effect	–3.87	–5.76	–1.99	–3.91	–5.97	–1.85
		*Indirect effect*	–***0.64***	–***1.68***	–***0.06***	–*0.60*	–*1.99*	*0.18*

*CI, confidence interval; Meaningful mediation effects in boldface.*

*The total effect is the effect of Impairment Status (X) on Language (Y), excluding Sustained Attention (M).*

*The direct effect is the effect of Impairment Status (X) on Language (Y), controlling for Sustained Attention (M).*

*The indirect effect is the effect of Impairment Status (X) on Language (Y) through Sustained Attention (M).*

#### Effect of LI in the Bilingual Group

**Table 6** presents the results of the mediation analyses investigating the effects of auditory and visual sustained attention on the relation between Impairment Status and language outcomes in the bilingual group of children. Bootstrapped tests (5.000 – bias-corrected), and confidence intervals were again used to determine whether sustained attention significantly reduced the effect of Impairment Status on vocabulary and morphology. The results from the analyses in the bilingual group suggest that both auditory and visual sustained attention act as partial mediators of the effect of LI on language abilities in both language domains and at both time points. At wave 2, the index of mediation was larger for vocabulary (auditory: *b* = -0.10, 95% CI [-0.25, -0.02]; visual: *b* = -0.14, 95% CI [-0.29, -0.04]) than morphology (auditory: *b* = -0.08, 95% CI [-0.20, -0.02]; visual: *b* = -0.08, 95% CI [-0.22, -0.004]), but there was substantial overlap in confidence intervals, indicating that reliable differences cannot be assumed. At wave 3, the reverse pattern was seen in the analyses with visual sustained attention (vocabulary: *b* = -0.09, 95% CI [-0.23, -0.01]; morphology: *b* = -0.11, 95% CI [-0.25, -0.02]). In the analyses with auditory sustained attention, the index was the same for both domains at wave 3 (vocabulary: *b* = -0.08, 95% CI [-0.20, -0.01]; morphology: *b* = -0.08, 95% CI [-0.22, -0.01]). Correlations between children’s language and sustained attention skills and visual representations of the mediation models are provided in the Supplementary Table 2 and Figures 5–8, respectively.

**Table 6:** Mediation effects of auditory and visual sustained attention on the relation between Impairment Status and language outcomes in the bilingual group of children.

			Auditory			Visual		
				95% CI			95% CI	
Language outcome	Wave	Effect	*b*	Lower	Upper	*b*	Lower	Upper
Vocabulary	2	Total effect	–9.82	–16.22	–3.42	–9.82	–16.22	–3.42
		Direct effect	–7.08	–13.43	–0.73	–6.15	–12.57	0.26
		*Indirect effect*	–***2.74***	–***6.67***	–***0.62***	–***3.67***	–***8.15***	–***0.94***
	3	Total effect	–10.02	–16.36	–3.68	–10.02	–16.36	–3.68
		Direct effect	–7.95	–14.37	–1.53	–7.71	–14.34	–1.08
		*Indirect effect*	–***2.07***	–***5.55***	–***0.29***	–***2.31***	–***6.25***	–***0.26***
Morphology	2	Total effect	–4.44	–6.48	–2.40	–4.44	–6.48	–2.40
		Direct effect	–3.76	–5.82	–1.70	–3.67	–5.80	–1.55
		*Indirect effect*	–***0.68***	–***1.83***	–***0.13***	–***0.77***	–***2.17***	–***0.03***
	3	Total effect	–5.57	–7.77	–3.38	–5.57	–7.77	–3.38
		Direct effect	–4.77	–6.97	–2.57	–4.48	–6.71	–2.25
		*Indirect effect*	–***0.80***	–***2.26***	–***0.14***	–***1.09***	–***2.75***	–***0.23***

*CI, confidence interval; Meaningful mediation effects in boldface.*

*The total effect is the effect of Impairment Status (X) on Language (Y), excluding Sustained Attention (M).*

*The direct effect is the effect of Impairment Status (X) on Language (Y), controlling for Sustained Attention (M).*

*The indirect effect is the effect of Impairment Status (X) on Language (Y) through Sustained Attention (M).*

## Discussion

The present study aimed to investigate the effects of an inborn LI and bilingualism on children’s language proficiency over time. Moreover, we addressed the question why this child-internal and child-external factor, respectively, produce overlap in children’s language profiles (e.g., Paradis, 2005). For the latter, we hypothesized that the language difficulties of children with LI stem from auditory sustained attention deficits, leading to incomplete processing of incoming language. As Leonard (2014) mentioned, “cases of incomplete processing are assumed to be the functional equivalent of reductions in input frequency” (p. 289), which draws a parallel between the origins of the language difficulties of children with LI and bilingual children, whose language skills are influenced by the distributed nature of their language input (Hoff et al., 2012). Two core language domains, i.e., vocabulary and morphology, were chosen as our outcome variables, as these are known to be affected by LI (Krok and Leonard, 2015; Rice and Hoffman, 2015) as well as by reduced input as a result of bilingualism (Scheele et al., 2010; Blom et al., 2012).

With a four-group design, including monolingual and bilingual children with and without LI, we first examined the effects of LI and bilingualism on children’s language development in Dutch. Vocabulary and morphology were assessed longitudinally and the results showed that, on both language domains and at each time point, the TD children outperformed the children with LI and the monolingual children outperformed the bilingual children. These findings correspond to previous work that identified persistent language delays of both children with LI (Rice, 2012; Rice and Hoffman, 2015) and bilingual children (Cobo-Lewis et al., 2002; Farnia and Geva, 2011; Paradis et al., 2016). However, we also found important differences in the way in which LI and (reduced input due to) bilingualism influenced a child’s language development. Effects of LI on vocabulary and morphology were large and remained stable over time, as expected (Rice, 2012). The effect of bilingualism on morphology also remained stable over time, likely due to a number of irregular items in our morphology task which have a low type frequency and are typically acquired at a late age (see, Boerma et al., 2017), but this effect was considerably smaller in magnitude than the effect of LI. Moreover, the difference in vocabulary size between the monolingual and bilingual children diminished over time, like in Farnia and Geva (2011). Despite persistent language delays in both groups, the most extensive overlap between the language profiles of the children with LI and bilingual children was thus evident on vocabulary in early (pre)school years. Future longitudinal research covering a longer period of time is needed to examine whether the overlap further reduces in later developmental stages.

To understand the source of this overlap, we furthermore investigated the effects of LI and bilingualism on children’s auditory and visual sustained attention skills, and explored the role of sustained attention in explaining the effects of LI on children’s language outcomes. In accordance with the meta-analysis of Ebert and Kohnert (2011), we found that the children with LI had a weaker ability to maintain their attention to the auditory and visual stimuli of the CPT than the TD children. Contrary to our predictions, the children with LI did not have more extensive problems with the auditory than the visual stimuli. Instead, the monolingual children with LI, like their monolingual TD peers, showed the reverse pattern, with a better performance on the auditory component of the CPT. This finding may be related to the integrated set-up of our task, in which auditory and visual stimuli were interspersedly presented during a prolonged period of time. To accurately respond to the visual targets and distractors, children were required to stay focused on the computer screen, whereas a quick look in another direction did not necessarily affect responses to auditory stimuli. Interestingly, this task effect did not influence the sustained attention performance of the bilingual children, both TD and LI, whose response sensitivity on the two modalities did not differ. It would be worthwhile to examine whether the use of a different sustained attention measure, with separate blocks of only visual or only auditory stimuli, would show the same results. We will come back to the discrepancy between the monolingual and bilingual children when discussing the outcomes of the mediation analyses.

While the results showed that LI was associated with weak sustained attention, no effect of bilingualism was found. Monolingual and bilingual participants scored equally well on the auditory and visual components of the CPT. Previous work reported a bilingual advantage on different attention measures (e.g., Bialystok, 1999; Engel de Abreu et al., 2012), but, to our knowledge, the current study is the first to specifically investigate sustained attention in bilingual children. Although Krizman et al. (2012) found better performance of bilingual adults in comparison with monolingual adults on a task targeting sustained attention, other adult studies failed to find this specific advantage (Bialystok et al., 2008; Bak et al., 2014). There are several factors that have been shown to moderate the effect of bilingualism on attention (and other aspects of cognition), which may explain the mixed findings in the literature and the absent positive effect of bilingualism in the current study. For example, a number of studies have shown that cognitive advantages are limited to bilinguals who are proficient in both languages (Carlson and Meltzoff, 2008; Poarch and van Hell, 2012; Weber et al., 2016) or emerge as an effect of growing bilingual proficiency (Blom et al., 2014; Crivello et al., 2016). It may thus be that the language proficiency of the bilingual children in our sample was not sufficiently strong for cognitive advantages to develop. In addition, it is also conceivable that bilinguals benefit from their bilingual language experience on certain cognitive measures, but not on others, as Bialystok et al. (2008) argue. Although common measures for sustained attention (including the measure used in the present study) require a degree of response inhibition, they involve simple stimuli and a rule dictating when to respond or refrain from responding. In contrast, measures such as the Simon or Stroop task, which also tap into attentional processing and on which a bilingual advantage has traditionally been found, use complex stimuli with multiple features that include a salient conflict (direction vs. position or word vs. color). Such conflict-monitoring is trained by interactions in bilingual contexts, explaining why a bilingual benefit may be limited to tasks that require substantial conflict resolution (for an elaborate discussion, see Bialystok et al., 2008). Nevertheless, even on those measures that require substantial conflict resolution, bilingual advantages are not always found (e.g., Antón et al., 2014; Duñabeitia et al., 2014), indicating that it is yet unclear under which specific conditions a bilingual benefit emerges.

To explore relations between the poor language abilities and the poor sustained attention skills of children with LI, we performed mediation analyses. Results showed that auditory sustained attention mediated the effect of LI on children’s language outcomes. This effect was stable, emerging on vocabulary and morphology, at wave 2 and 3, in the monolingual and bilingual group. These findings are in line with previous research that indicated positive associations between language and sustained attention in children with LI (Montgomery, 2008; Montgomery et al., 2009; Duinmeijer et al., 2012; Ebert et al., 2012, 2014; Blom and Boerma, 2016; Jongman et al., 2016). Although we hypothesized that sustained attention effects would be more pronounced on vocabulary than morphology, as a result of their susceptibility to input effects (Chondrogianni and Marinis, 2011), reliable differences between the two language domains were not found. As was mentioned before, this may be due to the complex irregular structures included in our morphology task (see, Gathercole, 2010; Paradis, 2010a). The inclusion of only regular items could possibly lead to different results and is an interesting venue for future research. Contrary to auditory sustained attention, visual sustained attention did not act as a meaningful mediator of the effect of LI on monolingual children’s language skills. This contrast between the auditory and visual modality seems to confirm our hypothesis that the language difficulties of the monolingual children with LI reflect, at least in part, a domain-specific weakened ability to maintain attention to auditory information, leading to incomplete processing of incoming language input. Thus, while reductions in input frequency cause language delays in bilingual children, the functional equivalent may impair the language proficiency of children with LI, resulting in partially overlapping language profiles.

In contrast to the monolingual children and contrary to our expectations, visual sustained attention did mediate the effect of LI on the vocabulary and morphology scores of bilingual children. Moreover, as mentioned before, there was also a discrepancy between the monolinguals and the bilinguals in terms of relative performance on the visual and auditory components of the CPT. While the two monolingual groups of children scored better on the auditory than the visual stimuli, the two bilingual groups performed equally well on both modalities. These discrepancies in our findings between the monolinguals and bilinguals may be related to research which showed that bilingual children attend more to visual speech cues in the environment in comparison with monolingual children, for whom these cues are redundant (Pons et al., 2015). In support of the complex task of dual language acquisition, bilinguals may exploit such visual information during social interactions more than monolinguals, enhancing the importance of visual sustained attention for successful language learning in bilingual contexts. If a child is less able to make use of these visual cues, due to poor visual sustained attention, this will hinder their acquisition of language, which is what the results from the present study suggest. Another possibility is that bilingual children rely more on orthographic learning than monolingual children to boost their second language skills. Several studies have shown that vocabulary learning in different populations, including bilinguals (Vadasy and Sanders, 2016) and children with LI (Ricketts et al., 2015), benefits from the presence of orthography. It may be that these orthographic facilitation effects are particularly strong in the context of dual language learning, explaining why visual sustained attention mediated the effect of LI on language in the bilingual group of children. Future research is necessary to investigate this hypothesis.

An alternative explanation for our findings could be that relations between children’s poor language abilities and poor sustained attention skills emerged as a result of a task effect. It may be that children need sustained attention to successfully complete the vocabulary and morphology task that we used to assess language competence. While this alternative interpretation cannot be ruled out, it does not accurately explain the discrepancy in our results between the auditory and the visual domain in the monolingual group of children. During both the vocabulary and the morphology task, children were required to maintain their attention to pictures as well as verbally presented words or sentences. If our findings were a mere reflection of task effects, both visual and auditory sustained attention would be expected to play a role. To investigate if attention influences children’s language performance in a task or also their language learning process, follow-up research could consider using measures from spontaneous speech data or using an experimental paradigm in which attention load is manipulated.

Although the findings from this study point to the importance of attention resources for the language proficiency of children with LI, they also indicate that sustained attention deficits only accounted for part of the effect of LI on children’s language skills. This is not surprising, as LI is a complex multifaceted disorder with no single underlying cause (Bishop, 2006). Future research is recommended to investigate multiple cognitive risk factors of LI, for example including both sustained attention and working memory, considering their individual contributions to the language deficit as well as how they interact. Moreover, future work needs to study the bidirectional relationships between language and cognition to further understand the behavioral profile of children with LI. The current study explored the effect of cognition on language, but reverse influences of language proficiency on cognition are also likely (e.g., Fuhs and Day, 2011; Kuhn et al., 2016) and could explain the co-occurrence of linguistic and non-linguistic weaknesses of children with LI (but see, Gooch et al., 2016). Finally, this study was limited by the heterogeneous sample of bilingual children, restricting the possibility to draw conclusions about specific groups. The bilingual children in our sample all learned Dutch as a second language, but varied considerably in degrees of exposure to Dutch and first language background. Such factors influence the severity and persistence of a bilingual child’s language delay (e.g., Paradis, 2010b; Blom et al., 2012), and are important to take into account in future work.

## Conclusion

The current study provided insight into the persistence and origins of the partially overlapping language profiles of bilingual children and children with LI. Our results showed that the language abilities of bilingual children and children with LI were persistently weaker than the language skills of monolingual and TD children, respectively. The overlap between the language profiles of bilingual children and children with LI was particularly large for vocabulary in early (pre)school years and diminished over time. Furthermore, our findings indicate that the overlap may be explained by the weakened ability of children with LI to maintain attention to the stream of linguistic information, interfering with how well incoming language is processed. While reductions in input frequency cause language delays in bilingual children, the functional equivalent, i.e., incomplete processing of input, may impair the language proficiency of children with LI. Next to auditory sustained attention, visual sustained attention also partly accounted for the language difficulties of bilingual children with LI, in contrast to their monolingual peers. These outcomes prompt further research on relations between LI, language skills and cognition in both monolingual and bilingual learning settings.

## Ethics Statement

This research was screened by the Standing Ethical Assessment Committee of the Faculty of Social and Behavioral Sciences at Utrecht University. Criteria were met and further verification was not deemed necessary. Parents of participants gave written informed consent in accordance with the Declaration of Helsinki.

## Author Contributions

All authors were involved in the conception and design of the study. TB wrote the manuscript and conducted the statistical analyses. PL, FW, and EB revised the draft for critical content.

## Conflict of Interest Statement

The authors declare that the research was conducted in the absence of any commercial or financial relationships that could be construed as a potential conflict of interest.
