# Supplementary material for: Language Proficiency and Sustained Attention in Monolingual and Bilingual Children with and without Language Impairment
Source: Front Psychol. 2017 Jul 21;8:1241. doi: 10.3389/fpsyg.2017.01241 (PMC5519625; doi:10.3389/fpsyg.2017.01241)
Supplement: Supplementary file 1 [file Supplementary_Material.pdf]

Supplemental materials – Correlations and Mediation analyses

Correlations

Table 1: Correlations between language outcomes and sustained attention in the monolingual group of children (significant correlations in boldface)

		**Typical Development**		**Language Impairment**	
	N	Auditory CPT	Visual CPT	Auditory CPT	Visual CPT
**PPVT Wave 2**	32	.07	.17	**.60****	.28
**PPVT Wave 3**	32	.17	.35†	**.48****	**.38***
**TAK Wave 2**	32	**.36***	.34†	.20	.07
**TAK Wave 3**	32	**.39***	**.40***	**.38***	.17

Note: **p < .01, *p < .05, †p < .10; CPT = Continuous Performance Task; PPVT = vocabulary; TAK = morphology.

Table 2: Correlations between language outcomes and sustained attention in the bilingual group of children (significant correlations in boldface)

		**Typical Development**		**Language Impairment**	
	N	Auditory CPT	Visual CPT	Auditory CPT	Visual CPT
**PPVT Wave 2**	32	.35†	**.48****	**.46****	**.51****
**PPVT Wave 3**	32	.23	.33†	**.42***	**.38***
**TAK Wave 2**	32	.33†	.23	.27	.30†
**TAK Wave 3**	32	.27	**.36***	**.39***	**.42***

Note: **p < .01, *p < .05, †p < .10; CPT = Continuous Performance Task; PPVT = vocabulary; TAK = morphology.

Mediation analyses

Effects of LI in the monolingual group – Auditory sustained attention

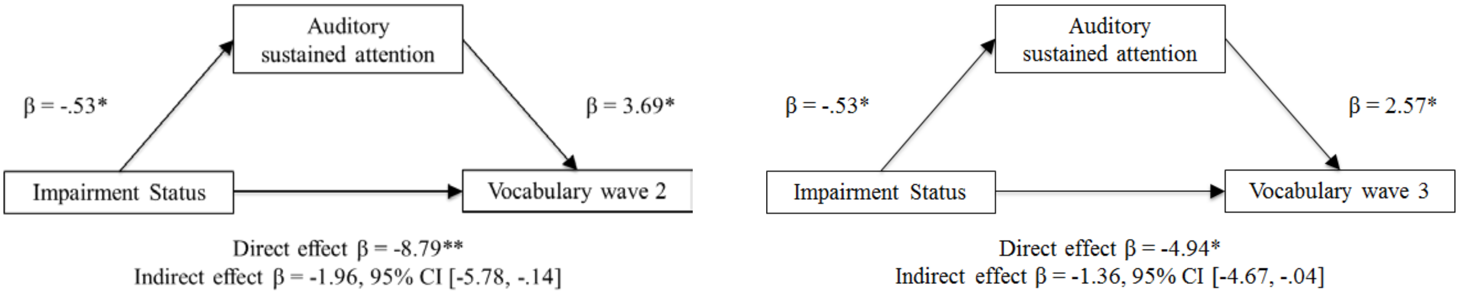

Figure 1: Mediation model with Impairment Status as independent variable, vocabulary outcomes at wave 2 and 3 as dependent variable, and auditory sustained attention as mediator.

Note: ***p* <.01; *p <.05; CI = Confidence Interval

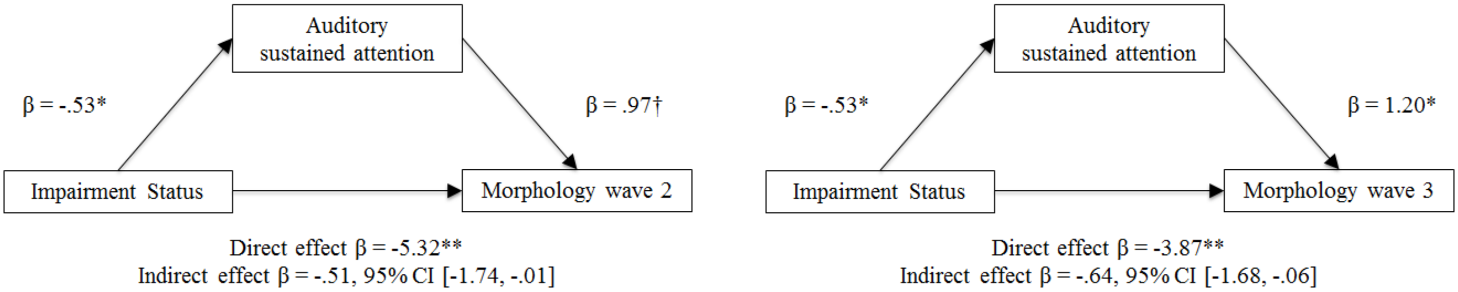

Figure 2: Mediation model with Impairment Status as independent variable, morphology outcomes at wave 2 and 3 as dependent variable, and auditory sustained attention as mediator.

Note: ***p* <.01; *p <.05; †*p* =.07; CI = Confidence Interval

Effects of LI in the monolingual group – Visual sustained attention

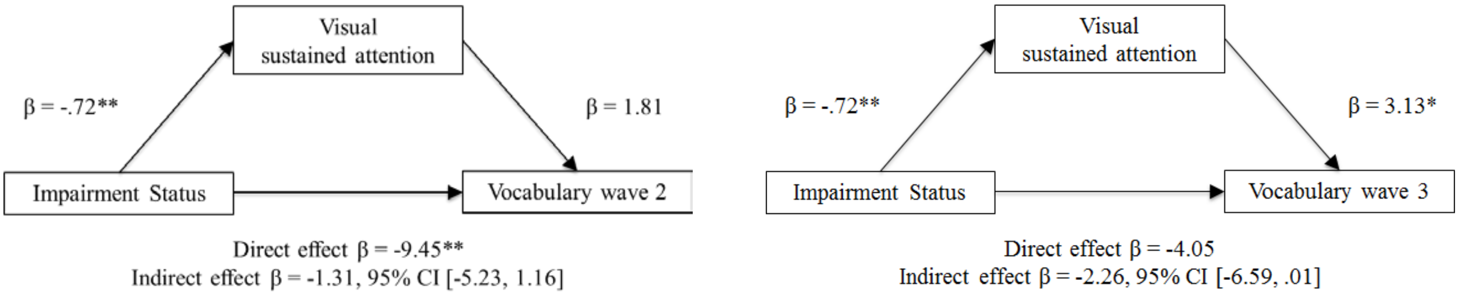

Figure 3: Mediation model with Impairment Status as independent variable, vocabulary outcomes at wave 2 and 3 as dependent variable, and visual sustained attention as mediator.
Note: ***p* <.01; *p <.05; CI = Confidence Interval

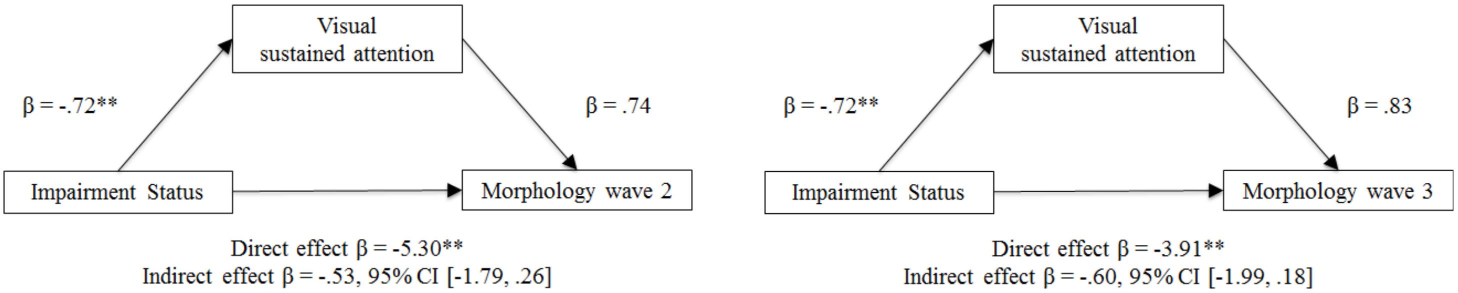

Figure 4: Mediation model with Impairment Status as independent variable, morphology outcomes at wave 2 and 3 as dependent variable, and visual sustained attention as mediator.
Note: ***p* <.01; *p <.05; CI = Confidence Interval

Effects of LI in the bilingual group – Auditory sustained attention

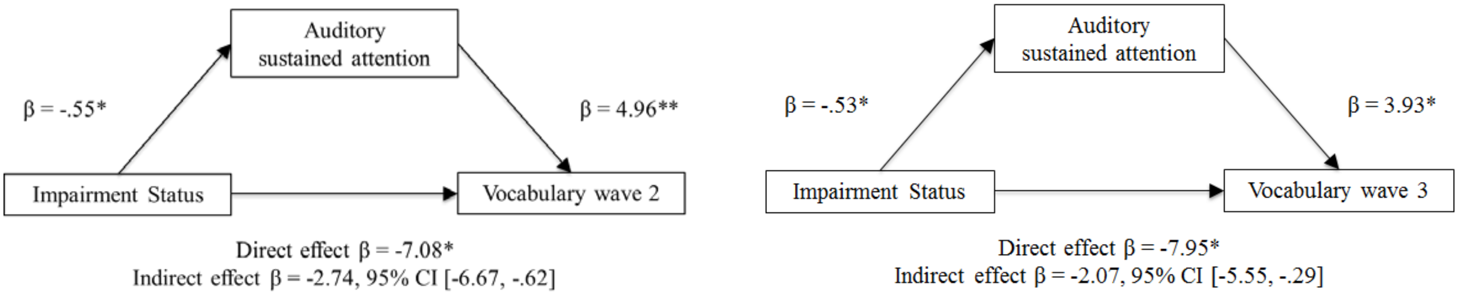

Figure 5: Mediation model with Impairment Status as independent variable, vocabulary outcomes at wave 2 and 3 as dependent variable, and auditory sustained attention as mediator.
Note: ***p* <.01; *p <.05; CI = Confidence Interval

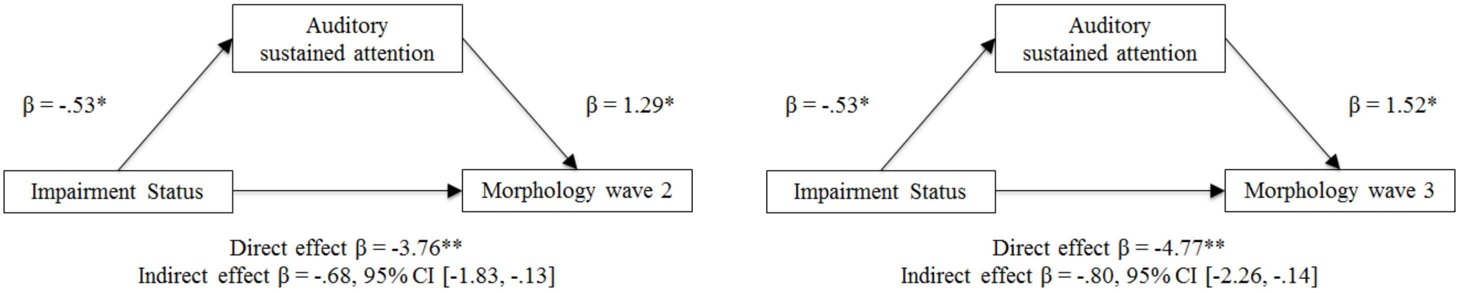

Figure 6: Mediation model with Impairment Status as independent variable, morphology outcomes at wave 2 and 3 as dependent variable, and auditory sustained attention as mediator.
Note: ***p* <.01; *p <.05; CI = Confidence Interval

Effects of LI in the bilingual group – Visual sustained attention

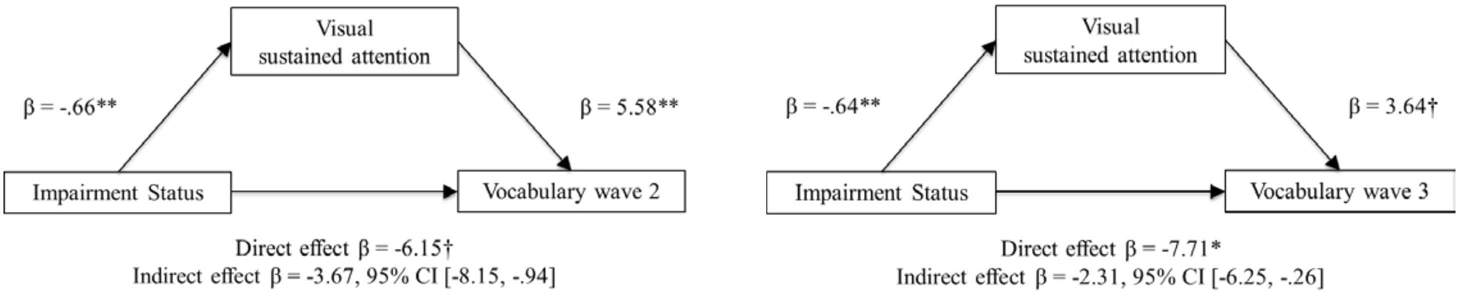

Figure 7: Mediation model with Impairment Status as independent variable, vocabulary outcomes at wave 2 and 3 as dependent variable, and visual sustained attention as mediator.
Note: ***p* <.01; *p <.05; †*p* =.06; CI = Confidence Interval

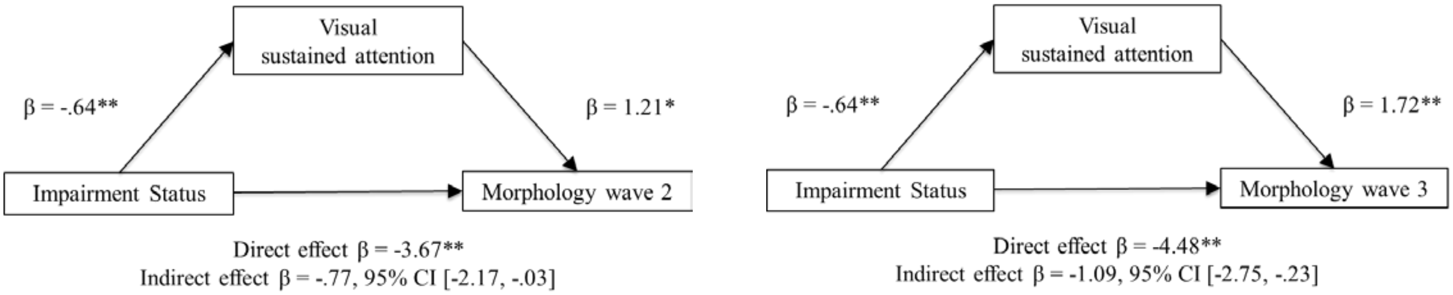

Figure 8: Mediation model with Impairment Status as independent variable, morphology outcomes at wave 2 and 3 as dependent variable, and visual sustained attention as mediator.
Note: ***p* <.01; *p <.05; CI = Confidence Interval
